# N-glycosylation of serum proteins for the assessment of patients with IgD multiple myeloma

**DOI:** 10.1186/s12885-017-3891-3

**Published:** 2017-12-21

**Authors:** Jie Chen, Meng Fang, Xiaoling Chen, Changhong Yi, Jun Ji, Cheng Cheng, Mengmeng Wang, Xing Gu, Quansheng Sun, Chunfang Gao

**Affiliations:** 1 Department of Laboratory Medicine, Shanghai Eastern Hepatobiliary Surgery Hospital, Second Military Medical University, 225 Changhai Road, Shanghai, China; 2 Department of Laboratory Medicine, Shanghai Jingan District Zhabei Central Hospital, 619 Zhonghuaxin Road, Shanghai, China; 3 Department of Hematology, Shanghai Jingan District Zhabei Central Hospital, 619 Zhonghuaxin Road, Shanghai, China

**Keywords:** IgD multiple myeloma, N-glycan profiling, Glycosylation, Biomarker, Diagnosis, Prognosis

## Abstract

**Background:**

Because glycosylation is one of the most common post-translational modifications of proteins and because changes in glycosylation have been shown to have a significant correlation with the development of many cancer types, we investigated the serum N-glycome used to diagnose, stage and evaluate the pathological outcomes in IgD multiple myeloma.

**Methods:**

Serum samples were available for 20 patients with IgD multiple myeloma, 41 patients with light chain multiple myeloma and 42 healthy control subjects. Serum N-glycans were released and analysed using DNA sequencer-assisted fluorophore-assisted capillary electrophoresis.

**Results:**

Characteristic changes were revealed in the serum N-glycome of IgD myeloma. In particular, three N-glycans (NG1(6)A2F, Peak3; NG1(3)A2F, Peak4; NA2FB, Peak7) showed increased clinical value. The best area under the ROC curve of NG1(6)A2F to diagnose IgD myeloma was 0.981, with a 95.0% sensitivity and 95.2% specificity, and that of NG1(3)A2F was 0.936, with a 95.0% sensitivity and 78.6% specificity. The best area under the ROC curve of NA2FB/NG1(3)A2F to differentially diagnose IgD myeloma versus light chain myeloma was 0.744, with a 95.3% sensitivity and 50.0% specificity. The level of NG1(3)A2F was correlated with the international staging system, while the higher abundance of NA2FB presented in IgD myeloma was predictive of a shorter progression-free survival.

**Conclusions:**

The advent of serum N-glycan signatures may play a role in the diagnosis, staging and prognosis of IgD myeloma and will serve as the foundation for a precision medicine approach to this rare subtype of multiple myeloma.

**Electronic supplementary material:**

The online version of this article (10.1186/s12885-017-3891-3) contains supplementary material, which is available to authorized users.

## Background

Multiple myeloma (MM) is a B-cell malignancy characterized by the expansion of malignant plasma cells within the bone marrow. IgD multiple myeloma (IgD MM) is an uncommon variant of MM, accounting for approximately 1% to 2% of myeloma cases. IgD MM has been characterized by relatively younger patients, male predominance, extramedullary involvement, osteolytic lesions, a λ chain bias, Bence Jones proteinuria, renal dysfunction and a poor prognosis [1–4].

Glycosylation is the stepwise procedure of the covalent attachment of oligosaccharide chains to proteins or lipids that may influence biological activity, stability, pharmacokinetics, and antigenicity, among other activities [5]. Oligosaccharides may be present as N-linked or O-linked forms. Variations in N-linked oligosaccharides are involved in many pathological conditions such as chronic diseases and cancers [6–9]. Recently, Mittermayr S et al. [10] have demonstrates the feasibility of mapping glycan modifications on the IgG molecule and provided the principle that differential IgG glycosylation patterns can be successfully identified in monoclonal gammopathy of undetermined significance (MGUS), smoldering multiple myeloma (SMM), active MM, complete-response post-treatment MM and relapse MM. Kovacs Z et al. [11] have indicated 12 N-glycan features showed statistically significant differences among various stages of MM in comparison to the control at the serum level, while only six features were identified with similar significance at the immunoglobulin level, including the analysis of the partitioned Fc fragment as well as the Fab κ and Fab λ chains. Consequently, the identification and reliable quantification of glycan biomarkers from serum samples may enable non-invasive cancer screening for diagnosis and prognosis. Over the past decade, the development of high-throughput N-glycan profiling methods (e.g. DNA sequencer-assisted fluorophore-assisted capillary electrophoresis, i.e., DSA-FACE) has enabled researchers to pursue the role of glycomics as potential disease biomarkers [12].

The findings related to the glycosylation of proteoglycan syndecan-1 (CD138) emphasize its relevance to growth, metastasis, prognosis and targeted therapy of MM. Syndecan-1 acts as a positive regulator of many effective molecules important for myeloma growth and survival, such as a proliferation-inducing ligand (APRIL), epidermal growth factor (EGF) family members, insulin-like growth factor (IGF), insulin-like growth factor binding proteins (IGFBP) or hepatocyte growth factor (HGF) and an inhibitor of human heparanase or heparinase III [13–18]. Until now, little was known regarding the changes in N-glycans in IgD MM. We recently demonstrated that the serum N-glycan profile models distinguished patients with light chain multiple myeloma (LCMM) from healthy control subjects (CTRs) and differentiated LCMM from other subtypes of multiple myeloma (IgG and IgA) [19].

One distinctive feature of IgD MM is the smaller size or absence of the monoclonal protein spike [20], which leads to false-negative results by conventional techniques (e.g., serum protein electrophoresis [SPE] and immunofixation electrophoresis [IFE]). Pandey S [21] reported that the serum concentrations of IgG and IgA were 1020 mg/dL to 1460 mg/dL and 210 mg/dL to 350 mg/dL, respectively, much higher than the level of IgD in serum (0 to 10 mg/dL). Thus, the undetectability or low amount of serum IgD may cause diagnostic errors (e.g., osteoclasia or impaired renal function) and a delayed diagnosis (e.g., diagnosed first as LCMM or non-secretory MM). The purpose of this study was to examine the serum N-glycan levels in patients with IgD MM and identify whether N-glycans could serve as diagnostic and prognostic markers for this condition.

## Methods

### Patients and serum samples

Representative serum samples from MM patients recruited during a 2-year period (March 2010 to April 2012) at Shanghai Jingan District Zhabei Central Hospital were studied, excluding those with the IgG or IgA subtype. Additionally, serum samples from healthy donors who were deemed free of diseases after a physical examination were collected. The 61 MM patients consisted of 20 IgD MM patients and 41 LCMM patients, and 42 CTRs were evaluated. All MM patients were defined based on the International Myeloma Working Group (IMWG) criteria 2003 [Clonal bone marrow plasma cells ≥10%, presence of serum or urinary monoclonal protein (except in patients with true non-secretory MM), and evidence of end-organ damage that can be attributed to the underlying plasma cell proliferative disorder] and were staged according to the international staging system (ISS) and Durie–Salmon staging system after a confirmed diagnosis [22, 23]. Both newly diagnosed MM patients and confirmed MM patients who were undergoing routine chemotherapy were included in the study. MM patients who had serious infectious diseases, acute or chronic inflammatory diseases, history of another malignant cancer besides MM, and drug abuse were excluded. The serum samples from newly diagnosed and treated patients were obtained before chemotherapy initiation and before the next chemotherapy session, respectively. Based on a computerized database and medical records of all patients seen at the hospital, the progression-free survival (PFS) and overall survival (OS) of 20 patients with IgD MM were monitored. Blood was collected using a standard protocol and serum samples were separated by centrifugation at 3000 rpm for 10 min, followed by storage at −80 °C until analysis. Nine patients were newly diagnosed and 11 were already treated in the IgD MM group. All the patients were already symptomatic or became symptomatic during follow-up. Next, the symptomatic patients were treated with conventional modalities. The median follow-up for IgD MM patients was 31.5 months.

### Laboratory tests and clinical features

The haematological index of the haemoglobin (Hb) level was determined using an automatic cell counter and associated reagent (Sysmex XZ-2100D Cell Counter; Sysmex, Kobe, Japan; Sysmex diagnostic reagents). Biochemical indexes, such as total protein (TP), albumin (ALB), urea (BUN) and creatinine (CREA) were measured using an automatic chemical analyser and associated reagent (ADVIA 2400 Analyzer; Siemens AG, Munich, Germany; Siemens diagnostic reagents). SPE and IFE were run using a semi-auto electrophoresis system (Sebia HYDRASYS2 electrophoresis system; Sebia, Tours, France). Serum and urine light chain levels were analysed on a Dade Behring BNII nephelometer (Siemens AG; Siemens diagnostic reagents).

Clinical features, such as bone lesion and extramedullary infiltration were documented from the medical records of patients with IgD MM.

PFS was calculated from the start of the first treatment to disease progression or death from any cause, or the date on which the patient was last known to be in remission. OS was calculated from the start of first treatment to the date of death or the date on which the patient was last known to be alive. For the analysis of the correlation between N-glycan abundance and survival, PFS or OS, the patients were divided into two groups: one group had a specific N-glycan structure level less than the median level, and the other group had a specific N-glycan structure level above the median level.

### Serum protein N-glycan profiling

The N-glycans present on the serum proteins were analysed using DSA-FACE technology as described previously [12, 24]. Briefly, the N-linked glycans were denatured and released from serum glycoproteins by adding the peptide N-glycosidase-F (PNGaseF) (New England Biolabs, Boston, MA) in 2 μl of serum. Thereafter, N-glycans were labelled with APTS (8-aminonaphtalene-1, 3, 6-trisulphonic acid) (Invitrogen, Carlsbad, CA). Sialic acid was removed using *Arthrobacter ureafaciens* sialidase (Roche Bioscience, Palo Alto, CA) and the processed samples were analysed using a capillary electrophoresis-based ABI3500 Genetic Analyzer (Applied Biosystems, Foster city, CA). Twelve obvious N-glycan peaks detected in all serum samples were analysed using the GeneMapper v4.1 software (Applied Biosystems). The abundance of each N-glycan peak was described by normalizing its height to the sum of the heights of all 12 peaks. Before applying DSA-FACE method to discover N-glycan biomarkers for IgD myeloma, we have evaluated the feasibility of this method, such as test reproducibility. The coefficient of variation (CV) value of run-to-run was less than 15% for each N-glycan marker. In addition, a pooled serum was aliquoted as the standard sample, and it was analyzed in each experiment to ensure the stability of the system and the reliability of the results (Additional file 1: Table S1).

### Statistical analysis

All quantitative variables are expressed as means ± standard deviation (SD). Quantitative variables were compared using Student’s t test, ANOVA or nonparametric tests. After one-way ANOVA, the LSD (least significance difference) test was applied for pair-wise comparison between the three groups. Pearson’s coefficients of correlation and their associated probabilities (*P*) were used to evaluate the relationship between the peak values and other independent parameters. The diagnostic or differentially diagnostic performance of a single marker was evaluated by receiver operating characteristic (ROC) curve analysis and the area under the curve (AUC). The sensitivity, specificity, positive predictive value (PPV), negative predictive value (NPV), and accuracy were calculated using cut-off values optimally selected based on the ROC curves. Survival curves were estimated according to the Kaplan-Meier method. Differences between survival curves were tested for statistical significance using the two-sided log-rank test. All reported *P* values were 2-tailed, and *P* values <0.05 were evaluated as statistically significant. Statistical analyses were performed using SPSS16.0 for Windows software (SPSS, Chicago, IL).

## Results

### Characteristics of N-glycome profiling in IgD MM

Twenty patients with IgD MM were identified, and the patient characteristics are presented in Table 1.

**Table 1:** Baseline characteristics of patients with IgD MM, LCMM and healthy CTRs

M protein type	IgD MM	LCMM	Healthy CTR
	(κ 1, λ 19)	(κ 19, λ 22)	(*n* = 42)
Male (%)	12 (60.0%)	26 (63.4%)	25 (59.5%)
Age	56.75 ± 9.51	58.17 ± 12.14	59.14 ± 5.90
ISS stage I	4 (20.0%)	11 (26.8%)	/
ISS stage II	5 (25.0%)	3 (7.3%)	/
ISS stage III	11 (55.0%)	27 (65.9%)	/
Durie Salmon stage IIIA	16 (80.0%)	/	/
Durie Salmon stage IIIB	4 (20.0%)	/	/
Haemoglobin	89.50 ± 20.61	100.28 ± 28.46	146.34 ± 9.42
Total protein	62.95 ± 8.83	60.88 ± 7.89	76.24 ± 3.87
Albumin	36.33 ± 4.60	39.23 ± 5.51	47.17 ± 2.18
Serum FLC ratio	2.62 ± 1.74	/	/
Urine FLC ratio	204.47 ± 262.98	/	/
SPE positive (%)	13 (65.0%)	11 (26.8%)	/
IFE positive (%)	18 (90.0%)	23 (56.1%)	/
Bone lesions (%)	14 (70.0%)	/	/
Extramedullary infiltration (%)	3 (15.0%)	/	/

Note: Quantitative data are expressed as the means ± standard deviation; qualitative data are expressed as n (%);/represents no relevant data documented

Abbreviations: *ISS* international staging system, *SPE* serum protein electrophoresis, *IFE* immunofixation electrophoresis, *FLC* free light chain (including 15 serum samples and 16 urine samples in patients with IgD MM)

**Fig. 1:**
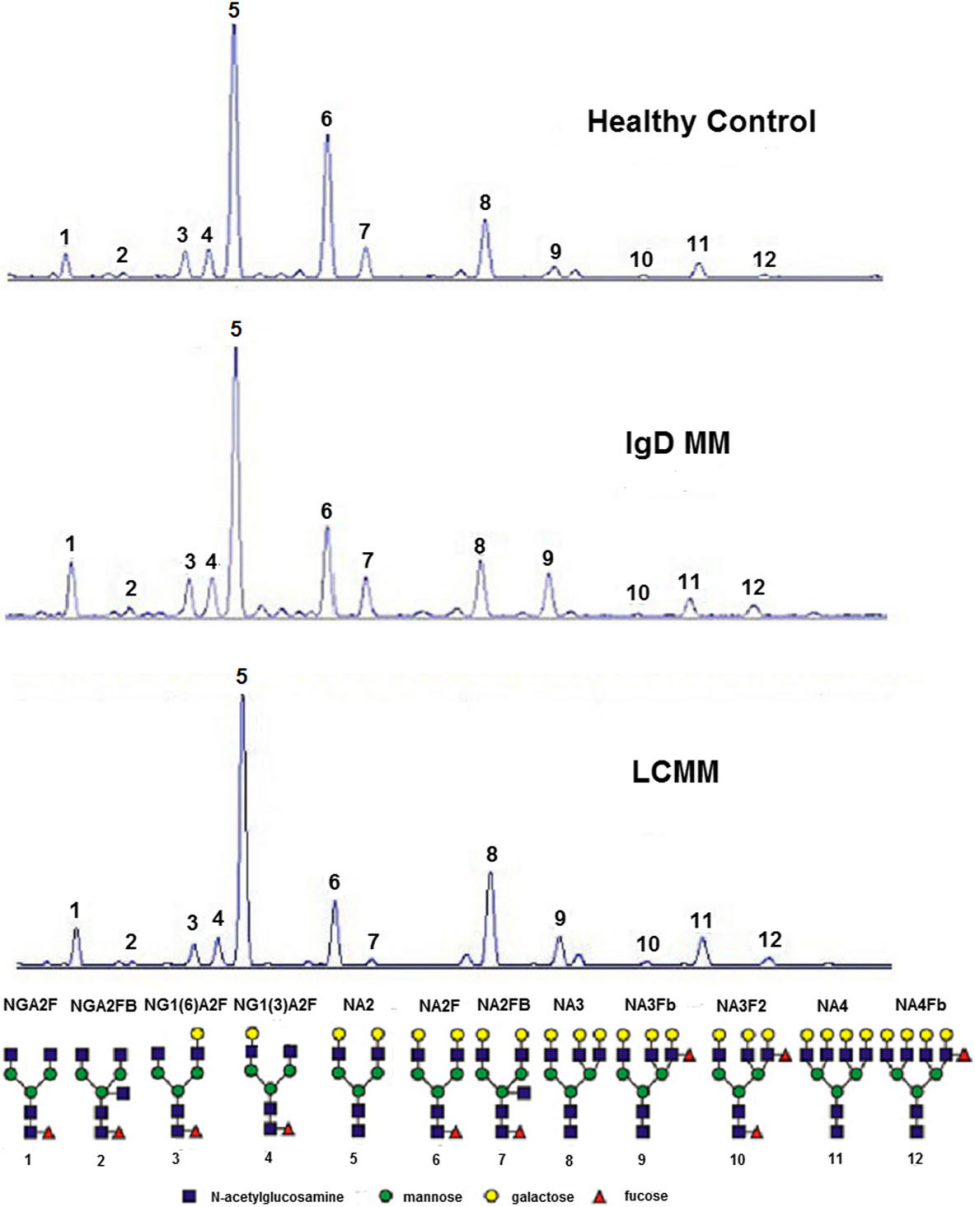
Characteristic N-linked glycan profile from total serum proteins. Twelve peaks were confirmed in the IgD MM, LCMM and healthy control groups. The structures of the corresponding N-glycan peaks are presented

As mentioned above, 12 N-glycan peaks were detected in all the serum samples (Fig. 1). The corresponding structure of each N-glycan peak was previously determined by Liu et al. and Bunz SC et al. [25–27]. The average abundance of the 12 N-glycan peaks in the IgD MM, LCMM and healthy CTR groups is shown in Table 2.

**Table 2:** Abundance of 12 N-glycan structures attached to serum proteins in IgD MM, LCMM patients and healthy CTRs

Structures	Healthy CTR	IgD MM	LCMM	*P1*	*P2*
	(*n* = 42)	(κ 1, λ 19)	(κ 19, λ 22)		
NGA2F	7.79 ± 1.94	5.77 ± 2.04	6.93 ± 3.00	<0.001	NS
NGA2FB	1.30 ± 0.45	1.06 ± 0.80	0.90 ± 0.54	NS	NS
NG1(6)A2F	6.32 ± 1.11	3.17 ± 1.09	3.44 ± 1.40	<0.001	NS
NG1(3)A2F	5.61 ± 1.22	3.53 ± 0.97	4.15 ± 0.84	<0.001	0.013
NA2	38.76 ± 3.06	44.07 ± 6.20	47.49 ± 6.43	0.001	NS
NA2F	20.14 ± 2.54	18.50 ± 9.56	14.76 ± 5.63	NS	NS
NA2FB	6.21 ± 1.40	4.85 ± 3.37	2.52 ± 2.40	NS	0.009
NA3	7.80 ± 1.96	9.84 ± 3.26	10.08 ± 3.51	0.016	NS
NA3Fb	2.68 ± 1.28	4.57 ± 3.14	5.04 ± 3.21	0.017	NS
NA3F2	0.34 ± 0.13	0.42 ± 0.16	0.46 ± 0.23	0.035	NS
NA4	1.69 ± 0.61	2.97 ± 0.95	3.01 ± 1.36	<0.001	NS
NA4Fb	0.47 ± 0.24	1.25 ± 0.89	1.22 ± 0.73	0.001	NS

Note: Quantitative data are expressed as the means ± standard deviation; *P1*: comparison between IgD MM patients and healthy CTR

*P2*: comparison between IgD MM and LCMM patients

Abbreviations: *IgD MM* IgD multiple myeloma, *LCMM* light chain multiple myeloma, *CTR* control subject, *NS* non-significant

The levels of three complex-type biantennary N-glycans, the agalactosylated NGA2F, α(1,6)-arm monogalactosylated NG1(6)A2F and α(1,3)-arm monogalactosylated NG1(3)A2F (identified as peaks 1, 3 and 4, respectively) were significantly lower in the IgD MM patients than in the CTRs. Moreover, the levels of bigalactosylated biantennary glycan NA2 (peak5), together with all the triantennary and tetra-antennary glycans NA3, NA3Fb, NA3F2, NA4 and NA4Fb (defined as peaks 8, 9, 10, 11 and 12, respectively), were significantly higher in the IgD MM patients than in the CTRs.

Next, we compared the IgD MM and LCMM groups because their serum N-glycomic profiles were similar to those of the CTRs. The 2 groups showed low levels of partial biantennary structures and high levels of NA2, triantennary and tetra-antennary glycan structures. The increase in multi-antennary peaks may affect the decreased concentration of IgG present in high abundance in the human circulation and the peaks represent predominantly biantennary complex-type structures. Notably, the significant decrease in NG1(3)A2F levels and prominent increase in NA2FB (peak7) levels were more evident in patients with IgD MM than in those with LCMM. In addition, none of the N-glycan peaks were significantly different between the newly diagnosed and treated IgD MM patients (Additional file 2: Table S2), and there were no differences between the kappa and lambda chain subjects with IgD MM because of the predominance of lambda chain (accounting for 95%).

### N-glycan biomarkers in different stages of IgD MM

**Fig. 2:**
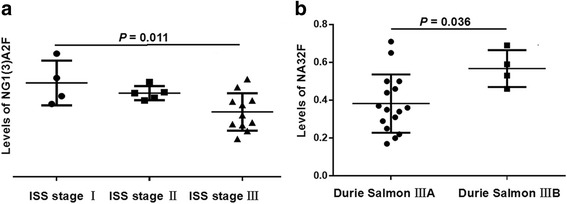
Comparison of the serum N-glycan structures according to different disease stage systems in IgD MM patients. **a** The tendency towards lower levels of NG1(3)A2F (peak4) was present in patients with a higher ISS stage, and levels of NG1(3)A2F were significantly different between ISS stage I and ISS stage III. **b** The levels of NA3F2 (peak10) were significantly higher in Durie Salmon stage IIIB than in stage IIIA

Next, we evaluated the association between the levels of serum N-glycomic structures and different tumour stages. For this purpose, 4 ISS stage I, 5 ISS stage II and 11 ISS stage III IgD MM patients were recruited; additionally, all the patients with IgD MM were diagnosed with Durie Salmon stage III (including 16 IIIA and 4 IIIB patients). NG1(3)A2F levels differed significantly between ISS stage I and ISS stage III patients (*P* = 0.011, Fig. 2a). Moreover, the level of NA3F2 was significantly higher in patients with Durie Salmon stage IIIB than in those with stage IIIA (*P* = 0.036, Fig. 2b). Therefore, the lower trend of the NG1(3)A2F level was related to a higher ISS stage, and a higher level of NA3F2 was associated with a greater probability of Durie Salom stage IIIA to IIIB.

### Association of the N-glycan biomarkers and laboratory parameters in IgD MM

**Fig. 3:**
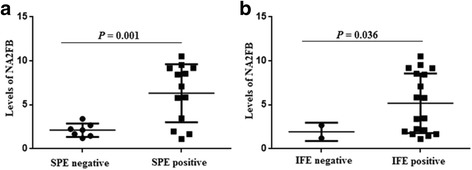
Comparison of the levels of NA2FB (peak7) between the SPE- and IFE-positive and -negative IgD MM patients. **a** Different levels of NA2FB were found between the SPE-positive and -negative groups. **b** Different levels of NA2FB were shown between the IFE-positive and -negative groups

Until now, SPE and IFE were the preferred methods of detection of monoclonal proteins in IgD MM patients. Therefore, we compared the levels of N-glycan peaks between the negative and positive groups. We found that, for SPE and IFE, the levels of NA2FB were significantly higher in the positive group compared with the negative group (*P* = 0.001 and *P* = 0.036, respectively, Fig. 3). Moreover, other laboratory parameters, namely, haemoglobulin (Hb), platelets (PLT), serum/urine light chain ratio, urea (BUN), creatinine (CREA), total protein (TP) and albumin (ALB) were collected. The decreased N-glycan peaks in the IgD MM patients presented a negative association with the serum or urine light chain ratios and a positive association with Hb, such as NGA2F versus the urine light chain ratio; NG1(6)A2F or NG1(3)A2F versus Hb and the serum/urine light chain ratio (Table 3). Additionally, increased N-glycan peaks showed a negative correlation with TP and ALB, e.g., NA2 or NA4 versus TP and NA3F2 versus ALB (Table 3).

**Table 3:** Comparison between N-glycan markers and laboratory parameters in IgD MM patients

Correlations		NGA2F	NG1(6)A2F	NG1(3)A2F	NA2	NA3F2	NA4
Hb	*r*	0.242	0.531	0.642	0.233	−0.323	−0.241
(*n* = 20)	*P*	0.304	0.016^a^	0.002^a^	0.322	0.164	0.305
TP	*r*	−0.076	−0.085	−0.320	−0.604	−0.094	−0.520
(*n* = 20)	*P*	0.749	0.720	0.170	0.005^a^	0.695	0.019+
ALB	*r*	−0.298	−0.096	−0.033	−0.041	−0.503	−0.418
(*n* = 20)	*P*	0.203	0.688	0.889	0.863	0.024^a^	0.067
SLC ratio	*r*	−0.456	−0.531	−0.659	−0.353	−0.220	−0.176
(*n* = 15)	*P*	0.088	0.042^a^	0.007^a^	0.197	0.431	0.530
ULC ratio	*r*	−0.591	−0.549	−0.587	−0.411	−0.393	−0.166
(*n* = 16)	*P*	0.040^a^	0.015+	0.017^a^	0.114	0.132	0.583

Abbreviations: *Hb* haemoglobulin, *TP* total protein, *ALB* albumin, *SLC* serum light chain, *ULC* urine light chain

Using κ/λ ratio for IgD κ MM patients and λ/κ ratio for IgD λ MM patients

^a^represents N-glycan biomarkers with a significant correlation with corresponding laboratory indexes

### Performance of N-glycan biomarkers in the diagnosis and differential diagnosis of IgD MM

**Fig. 4:**
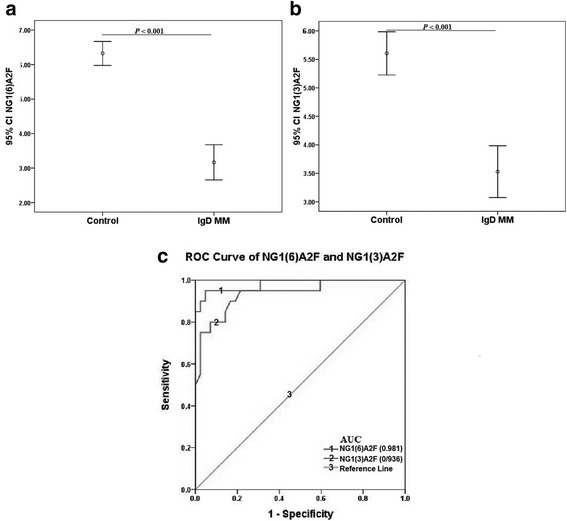
Diagnostic performance of N-glycan biomarkers in IgD MM. (**a**) and (**b**) The abundances of NG1(6)A2F (peak3) and NG1(3)A2F (peak4) were significantly decreased in patients with IgD MM than in CTRs. **c** ROC curves demonstrated the ability of serum NG1(6)A2F and NG1(3)A2F levels to diagnose IgD MM

To further evaluate the potential clinical usefulness of these 9 N-glycan constructions as diagnostic serum markers, we employed ROC curve analysis to assess these N-glycan markers. As shown in Fig. 4, NG1(6)A2F and NG1(3)A2F presented the best diagnostic performance, with AUCs of 0.981 (95% CI 0.949–1.013) and 0.936 (95% CI 0.869–1.003). The optimal cut-off value for NG1(6)A2F was 4.819 (sensitivity 95.0%, specificity 95.2%, accuracy 95.1%), and that for NG1(3)A2F was 4.640 (sensitivity 95.0%, specificity 78.6%, accuracy 86.8%) (Table 4). In this study, 7 SPE negative and 2 IFE negative IgD MM patients could be diagnosed properly by N-glycan markers NG1(6)A2F and NG1(3)A2F (Additional file 3: Table S3).F.

**Table 4:** Cut-off value, sensitivity, specificity, PPV, NPV and accuracy of N-glycan biomarkers in the diagnosis and differential diagnosis of IgD MM

Cut off value	Sensitivity (%)	Specificity (%)	PPV (%)	NPV (%)	Accuracy (%)
NG1(6)A2F (IgD MM vs Control)	95.0	95.2	95.2	90.5	95.1
(4.819)					
NG1(3)A2F (IgD MM vs Control)	95.0	78.6	81.6	94.0	86.8
(4.640)					
NA2FB/NG1(3)A2F (IgD MM vs LCMM)	95.3	50.0	65.6	91.4	72.7
(0.953)					

Abbreviations: *PPV* positive predictive value, *NPV* negative predictive value

**Fig. 5:**
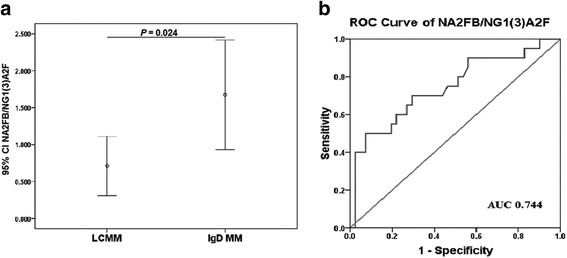
The differential diagnostic performance of N-glycan biomarkers in IgD MM. **a** The abundance of NA2FB/NG1(3)A2F (peak 7/peak 4) was significantly increased in patients with IgD MM compared with those with LCMM. **b** The ROC curve demonstrated the ability of the serum NA2FB/NG1(3)A2F levels to differentially diagnose IgD MM from LCMM

Because most of the IgG MM and IgA MM cases can be identified by SPE and IFE and the N-glycomic profiles of serum proteins were quite similar between the IgD MM and LCMM patients (as described above), we intended to differentially diagnose IgD MM from LCMM only. The ratio of NA2FB/NG1(3)A2F (peak7/peak4) was selected as the differential diagnostic marker for NA2FB levels were significantly higher in the IgD MM group than in the LCMM group, while NG1(3)A2F levels were significantly lower in the former than in the latter. NA2FB/NG1(3)A2F showed comparably differential diagnostic performance with an AUC of 0.744 (95% CI 0.605–0.883, Fig. 5). The optimal cut-off value for NA2FB/NG1(3)A2F was 0.953 (sensitivity 95.3%, specificity 50.0%, accuracy 72.7%) (Table 4).

In this study, one patient whose NA2FB/NG1(3)A2F level was 3.307 at the first-time diagnosis of LCMM was eventually confirmed with the subtype of IgD lambda 37 months later. Therefore, the increased level of NA2FB/NG1(3)A2F probably indicated the presence of IgD monoclonal protein, while IFE and SPE already showed a light chain-only or negative result.

### High levels of NA2FB correlate with a poor prognosis in IgD MM

**Fig. 6:**
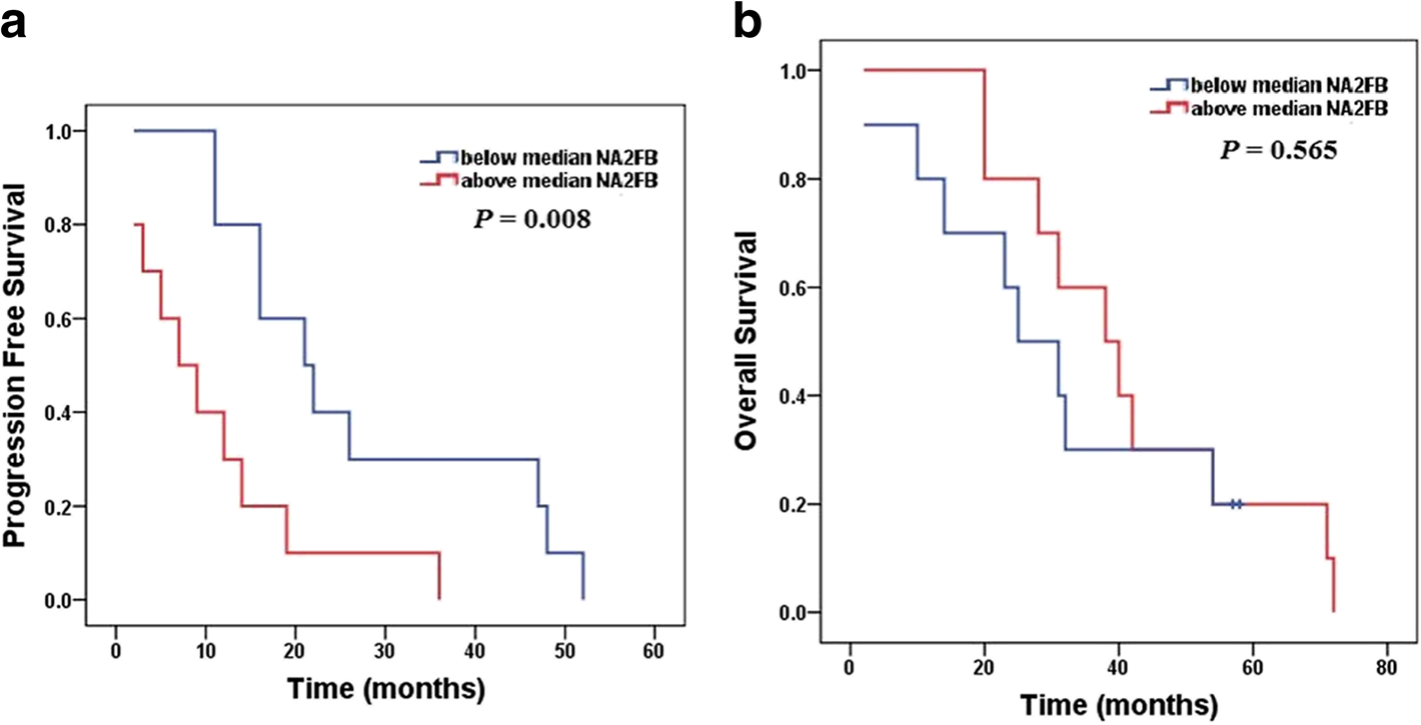
Survival curves for IgD MM using the Kaplan-Meier method and log-rank test. **a** Progression free survival (PFS) curves for patients with above median levels of NA2FB (red line, 10.9 months) and patients with below median levels of NA2FB (blue line, 27.0 months); (**b**) Overall survival (OS) curves for patients with above median levels of NA2FB (red line, 30.7 months) and patients with below median levels of NA2FB (blue line, 41.6 months)

To determine the correlation between the abundance of N-glycan markers and clinical outcome, we analysed the prognostic significance of N-glycan markers using Kaplan-Meier analysis. The median follow-up of the entire IgD MM patient cohort was 31.5 months. The comparison was executed between the above- and below-median concentrations of each N-glycan peak. Finally, as shown in Fig. 6, the progression free survival (PFS) time of IgD MM patients with above-median levels of NA2FB was significantly shorter than that of patients with below-median levels of NA2FB (*P* = 0.008). Additionally, the subjects with NA2FB levels above the median level exhibited a shorter overall survival (OS) time, and those with NA2FB levels below the median level showed a relatively longer OS time (30.7 and 41.6 months, respectively), although the difference was not statistically significant.

## Discussion

Glycosylation is a major post-translational modification of proteins for cellular function. Nearly half of human serum proteins, including secretory and membrane-bound proteins, are suggested to exhibit various N-linked glycosylation patterns [28]. Many glycan structures of proteins can influence various aspects of the biological behaviours of tumour cells during carcinogenesis [29–32]. Altered glycosylation patterns have long been recognized as hallmarks of cancer progression and metastasis [33–35]. The sensitive detection of serum N-glycosylation changes and abnormalities using the DSA-FACE technique developed in this study serve as a unique foundation for the development of diagnostic and prognostic N-glycan biomarkers for IgD MM. We found that the levels of NGA2F (peak1), NG1(6)A2F (peak3) and NG1(3)A2F (peak4) were significantly lower in the IgD MM patients than in the CTRs. In addition, the levels of NA2 (peak5), NA3 (peak8), NA3Fb (peak9), NA3F2 (peak10), NA4 (peak11) and NA4Fb (peak12) were prominently higher in the IgD MM patients than in the CTRs. These structural varieties may occur because of the altered levels of glycosyltransferases, glycosidases, and sugar nucleotide donors [36]. The increase in all triantennary and tetra-antennary N-glycans, with the decrease in agalactosylated and monogalactosylated structures, may provide more binding sites (galactose) for terminal sialic acid and activate related enzymes. Glavey SV et al. demonstrated the high expression of sialyltransferase ST3GAL6 (β-galactoside α-2, 3-sialyltransferase) influences homing and survival in MM [37]. Until now, certain sialyltransferase inhibitors may become novel therapeutic strategies targeting related abnormal glycosylation reported to suppress the malignancy, invasion, and metastasis of tumours [38–40].

We also identified a gradual downtrend of the NG1(3)A2F (peak4) levels from ISS stage I to ISS stage III in IgD MM patients. Significant differences occurred in the levels of NG1(3)A2F between the ISS stage I and ISS stage III groups. A significant increase was also found in the levels of NA3F2 (peak10) between Durie Salmon stage IIIB and IIIA patients. These serum N-glycomic changes indicate that the levels of NG1(3)A2F were strongly negatively correlated with ISS stage or IgD MM severity, and the levels of NA3F2 were positively correlated with renal insufficiency in patients with Durie Salmon stage III. Moreover, the levels of NGA2F, NG1(6)A2F or NG1(3)A2F were decreased more with the increased concentrations of the serum/urine light chain ratio and decreased levels of Hb. The levels of NA2, NA3F2 and NA4 were increased more with the decreased concentrations of TP or ALB. High levels of NA2FB were associated with SPE and IFE positivity. These results clearly demonstrated that the changes in some N-glycans are sensitive biomarkers for some typical syndromes in IgD MM, such as anaemia, abnormal secretion of monoclonal proteins and hypoproteinaemia.

As mentioned above, the main problem in diagnosing IgD MM is the uncertain appearance of an M spike in SPE, whether due to heavy chains or light chains. In this regard, Kuliszkiewicz-Janus M et al. reported an M spike in SPE only in 60% of IgD MM patients [20]. A similar observation was shown in this study with a 65% positive rate of SPE. The main reason is that IgD is a unique immunoglobulin with a serum concentration far below those of IgG, IgA, and IgM and has usually been ignored by routine tests. IgD accounts for 0.25% of the total serum immunoglobulins and has a half-life of 2.8 days [41]. The synthesis rate of IgD is at least 10 times lower than that of IgA, IgM, and IgG [42]. Arnaud P et al. [43] previously reported that only 31.5% of IgD was intravascular in patients with IgD MM. Therefore, the low concentration of IgD makes the detection result false-negative. All the patients with confirmed IgD MM in our study had Durie Salmon stage III. Some patients had been misdiagnosed with bone injury or had been diagnosed first with LCMM or non-secretory MM. Our study showed that the N-glycan biomarkers could overcome the limitation of measuring monoclonal IgD with conventional techniques (SPE or IFE). The sensitivity and specificity of NG1(6)A2F for the diagnosis of IgD MM were 95.0% and 95.2%, respectively, and the sensitivity and specificity of NG1(3)A2F were 95.0% and 78.6%, respectively. Both markers could avoid the false-negative SPE and IFE results in our study. Based on the diagnostic value of NG1(6)A2F in LCMM [19], we recommend NG1(3)A2F combined with NG1(6)A2F as diagnostic markers for IgD MM and NG1(3)A2F combined with NA2FB for LCMM. Additionally, the results of SPE and IFE, together with the quantitative determination of serum IgD and light chains using a nephelometer still need to be considered in the diagnosis.

Because of the similarity of the serum N-glycomic profile between IgD MM and LCMM, the differential diagnosis was focused on these two subtypes. The ratio of NA2FB/NG1(3)A2F was selected as the marker for the differential diagnosis with a sensitivity and specificity of 95.3% and 50.0%, respectively. Due to the presence of abnormal IgD monoclonal immunoglobulin in IgD MM, the increase in NA2FB and decrease in NG1(3)A2F may arise from the N-glycosylated characteristic of IgD. Arnold et al. [44] reported that 19% of N-glycans terminate in galactose residues, 50% of the glycans contain core fucosylation and 50% contain a bisecting GlcNAc at Asn-445 and Asn-496 of IgD. Therefore, whether the abnormal IgD leads to this specific change in IgD MM still needs further research in the future.

Moreover, the level of NA2FB was identified as an important prognostic factor in IgD MM. A higher level of NA2FB showed a prominent shorter PFS time than a lower level of NA2FB, but no statistical significance was found in OS between the higher and lower levels in the NA2FB group.

## Conclusions

In summary, the characterization of the N-glycomic profile was, for the first time, explained in patients with IgD MM. The findings presented here indicated a considerable diagnostic, staging and prognostic potential of the N-glycan biomarkers in this rare subtype of MM. We still need to emphasize the importance of disclosing the relevant molecular mechanisms underlying the unique composition of the N-glycan signatures in IgD MM.

## Additional files

Additional file 1: Table S1. The typical repetitive results of this internal standard. (DOCX 16 kb)

Additional file 2: Table S2. Abundance of N-glycan structures between newly diagnosed and treated patients with IgD MM. (DOCX 16 kb)

Additional file 3: Table S3. Abundance and cut-off values of NG1(6)A2F and NG1(3)A2F in 7 SPE negative and 2 IFE negative IgD MM. (DOCX 16 kb)

## Abbreviations

ALB: Albumin
APRIL: A proliferation-inducing ligand
APTS: 8-aminonaphtalene-1,3,6-trisulphonic acid
AUC: Area under the curve
BUN: Urea
CREA: Creatinine
CTRs: Healthy control subjects
CV: Coefficient of variation
DSA-FACE: DNA sequencer-assisted fluorophore-assisted capillary electrophoresis
EGF: Epidermal growth factor
Hb: Haemoglobin
HGF: Hepatocyte growth factor
IFE: Immunofixation electrophoresis
IGF: Insulin-like growth factor
IGFBP: Insulin-like growth factor binding proteins
IMWG: International Myeloma Working Group
ISS: International staging system
LCMM: Light chain multiple myeloma
MGUS: monoclonal gammopathy of undetermined significance
MM: Multiple myeloma
NPV: Negative predictive value
OS: Overall survival
P: Probability
PFS: Progression-free survival
PNGasF: Peptide N-glycosidase-F
PPV: Positive predictive value
ROC: Receiver Operating Characteristic
SD: Standard deviation
SMM: Smoldering multiple myeloma
SPE: Serum protein electrophoresis
TP: Total protein
