# Supplementary material for: N-glycosylation of serum proteins for the assessment of patients with IgD multiple myeloma
Source: BMC Cancer. 2017 Dec 21;17:881. doi: 10.1186/s12885-017-3891-3 (PMC5740902; doi:10.1186/s12885-017-3891-3)
Supplement: Supplementary file 1 — The typical repetitive results of this internal standard. (DOCX 16 kb) [file 12885_2017_3891_MOESM1_ESM.docx]

**Additional file 1**

**Table S1 The typical repetitive results of this internal standard**

| Variables | Mean | SD | CV(%) | Mean+2SD | Mean-2SD |
| --- | --- | --- | --- | --- | --- |
| NGA2F | 6.107 | 0.610 | 9.987 | 7.327 | 4.887 |
| NGA2FB | 0.899 | 0.119 | 13.215 | 1.136 | 0.661 |
| NG1(6)A2F | 5.140 | 0.446 | 8.683 | 6.032 | 4.247 |
| NG1(3)A2F | 5.834 | 0.414 | 7.093 | 6.662 | 5.007 |
| NA2 | 39.859 | 1.379 | 3.459 | 42.617 | 37.102 |
| NA2F | 23.535 | 0.701 | 2.979 | 24.937 | 22.133 |
| NA2FB | 5.671 | 0.226 | 3.988 | 6.123 | 5.219 |
| NA3 | 6.324 | 0.553 | 8.751 | 7.431 | 5.218 |
| NA3Fb | 2.985 | 0.213 | 7.147 | 3.412 | 2.558 |
| NA3F2 | 0.382 | 0.055 | 14.331 | 0.491 | 0.272 |
| NA4 | 1.671 | 0.211 | 12.633 | 2.093 | 1.249 |
| NA4Fb | 0.649 | 0.073 | 11.283 | 0.795 | 0.502 |

Note: Abbreviations: SD, standard deviation; CV, coefficient of variation
