# Supplementary material for: N-glycosylation of serum proteins for the assessment of patients with IgD multiple myeloma
Source: BMC Cancer. 2017 Dec 21;17:881. doi: 10.1186/s12885-017-3891-3 (PMC5740902; doi:10.1186/s12885-017-3891-3)
Supplement: Supplementary file 2 — Abundance of N-glycan structures between newly diagnosed and treated patients with IgD MM. (DOCX 16 kb) [file 12885_2017_3891_MOESM2_ESM.docx]

**Additional file 2**

**Table S2 Abundance of N-glycan structures between newly diagnosed and treated patients with IgD MM**

| Structures | Newly diagnosed | Treated | *P* |
| --- | --- | --- | --- |
|  | (n =9) | (n =11) |  |
| NGA2F | 5.91 ± 2.70 | 5.66 ± 1.45 | NS |
| NGA2FB | 1.05 ± 0.81 | 1.07 ± 0.83 | NS |
| NG1(6)A2F | 2.95 ± 1.41 | 3.34 ± 0.77 | NS |
| NG1(3)A2F | 3.38 ± 1.26 | 3.65 ± 0.70 | NS |
| NA2 | 41.24 ± 7.23 | 46.39 ± 4.26 | NS |
| NA2F | 21.92 ± 13.67 | 15.69 ± 2.21 | NS |
| NA2FB | 5.51 ± 3.22 | 4.33 ± 3.54 | NS |
| NA3 | 10.40 ± 4.32 | 9.38 ± 2.19 | NS |
| NA3Fb | 3.24 ± 1.78 | 5.65 ± 3.65 | NS |
| NA3F2 | 0.39 ± 0.18 | 0.45 ± 0.15 | NS |
| NA4 | 3.08 ± 1.03 | 2.88 ± 0.92 | NS |
| NA4Fb | 0.92 ± 0.54 | 1.52 ± 1.04 | NS |

Note: Quantitative data are expressed as means ± standard deviation; *P*: comparison between newly diagnosed and treated IgD MM,

Abbreviations: NS, non-significant
