# Supplementary material for: N-glycosylation of serum proteins for the assessment of patients with IgD multiple myeloma
Source: BMC Cancer. 2017 Dec 21;17:881. doi: 10.1186/s12885-017-3891-3 (PMC5740902; doi:10.1186/s12885-017-3891-3)
Supplement: Supplementary file 3 — Abundance and cut-off values of NG1(6)A2F and NG1(3)A2F in 7 SPE negative and 2 IFE negative IgD MM. (DOCX 16 kb) [file 12885_2017_3891_MOESM3_ESM.docx]

**Additional file 3**

**Table S3 Abundance and cut-off values of NG1(6)A2F and NG1(3)A2F in 7 SPE negative and 2 IFE negative IgD MM**

| Patient no. | SPE | IFE | NG1(6)A2F | NG1(3)A2F | Cut-off value |
| --- | --- | --- | --- | --- | --- |
| 1 | - | + | 4.57 | 4.57 | 4.819 for NG1(6)A2F  4.640 for NG1(3)A2F |
| 2 | - | + | 4.17 | 4.44 |  |
| 3 | - | - | 1.92 | 3.57 |  |
| 4 | - | - | 2.76 | 3.78 |  |
| 5 | - | + | 1.93 | 3.36 |  |
| 6 | - | + | 3.72 | 3.96 |  |
| 7 | - | + | 3.73 | 3.92 |  |

Note: -: negative result; +: positive result
